# Dispersal of *Aedes aegypti* in urban environments of Miami-Dade County, Florida

**DOI:** 10.1186/s13071-026-07445-7

**Published:** 2026-05-18

**Authors:** Hadian Iman Sasmita, Maday Moreno, Magic Vang, Chalmers Vasquez, Matthew DeGennaro, Rhoel R. Dinglasan, John-Paul Mutebi, John C. Beier

**Affiliations:** 1 Department of Public Health Sciences, Miller School of Medicine, University of Miami, Miami, FL USA; 2 Research Center for Radiation Process Technology, National Research and Innovation Agency (BRIN), Tangerang Selatan, Indonesia; 3 Miami-Dade County Mosquito Control Division, Miami, FL USA; 4 Department of Biological Sciences and Biomolecular Sciences Institute, Florida International University, Miami, FL USA; 5 Department of Infectious Diseases and Immunology, College of Veterinary Medicine, University of Florida, Gainesville, FL USA

**Keywords:** Dispersal, Mark–capture, Self-marking, Urbanization, Dengue, Miami-Dade, Florida

## Abstract

**Background:**

The dispersal of *Aedes aegypti* is influenced by anthropogenic features of urban environments and climatic factors. Miami-Dade County is undergoing rapid urban expansion, creating favorable conditions that support the proliferation of *Ae. aegypti*. In response to this heightened entomological risk and the continued occurrence of dengue transmission, strengthened vector control programs are essential. Effective mosquito control requires a detailed understanding of vector dispersal ecology. In this study, we aimed to estimate the dispersal parameters of local *Ae. aegypti* populations in two urban environments in Miami-Dade County.

**Methods:**

This dispersal study was conducted using rhodamine B self-marking mark–capture procedures. The self-marking unit consists of a rhodamine B sugar feeding apparatus for adult marking, an ovicup containing rhodamine B solution for larval marking, and CO_2_ lures. Capture was performed using BG-sentinel traps baited with CO_2_. Redlands and Opa-Locka, representing contrasting settings in population density, vegetation, and breeding habitats, were selected as the study sites. Dispersal capability, including distance traveled [minimum distance traveled (MinDT), maximum distance traveled (MaxDT), and mean distance traveled (MDT)] and flight range (FR) were calculated, and environmental factors influencing the captures of marked *Ae. aegypti* were analyzed.

**Results:**

A total of 216 and 36 marked specimens were captured in Redlands and Opa-Locka, respectively. The higher collections in Redlands were associated with higher vegetation coverage and the presence of a tire yard, while daily precipitation was negatively associated with marked specimen counts. The overall dispersal parameters were lower in an agricultural area in the suburban city of Redlands [MinDT = 10.07 m, MaxDT = 144.28 m, MDT = 63.65 m, 50% flight range (FR_50_) = 24.99 m, and 90% flight range (FR_90_) = 171.21 m], compared with those in a residential area in the city of Opa-Locka (MinDT = 28.60 m, MaxDT = 316.29 m, MDT = 194.03 m, FR_50_ = 131.83 m, FR_90_ = 329.58 m). The dispersal parameters estimated in this study were generally comparable to those reported in previous *Ae. aegypti* dispersal studies conducted in the USA.

**Conclusions:**

This study demonstrates the utility of rhodamine B self-marking procedures for measuring dispersal parameters and provides essential insight into *Ae. aegypti* dispersal that can enhance the precision and effectiveness of intervention programs.

**Graphical Abstract:**

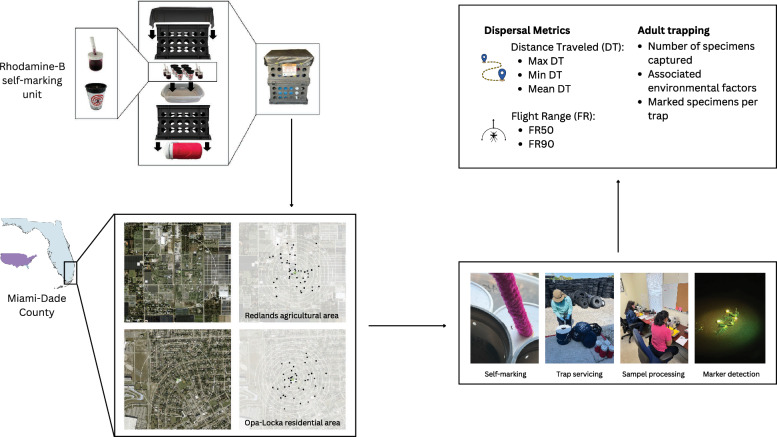

**Supplementary Information:**

The online version contains supplementary material available at 10.1186/s13071-026-07445-7.

## Background

Miami-Dade County has a long historical record of dengue virus (DENV) transmission and outbreaks associated with *Aedes aegypti* [1]. As the main vector for DENV, *Ae. aegypti* is present year-round and widespread throughout the county [2, 3], driven by rapid urbanization [4], favorable climatic conditions [5, 6], and widespread insecticide resistance [7]. In addition, the county’s role as a major gateway for human movement from southern endemic regions further increases DENV transmission risk [8]. A total of 2379 dengue cases, both locally acquired and travel-related, were reported in Miami-Dade County between 2010 and September 2025, the highest number recorded among all counties in the USA [9]. In response, mosquito control programs must be strengthened through continuous acquisition of vector biology baseline data.

Understanding the vector dispersal capability and ecology is a key baseline data component in strengthening vector control programs [10]. For instance, the flight distance of *Aedes* mosquitoes within a given ecological landscape can determine the appropriate area coverage for adulticide space spraying [11–13]. Although numerous studies have reported the dispersal of *Ae. aegypti*, data specific to Miami-Dade local populations are currently lacking. The dispersal of *Ae. aegypti* is generally considered short and is considerably influenced by anthropogenic factors such as local infrastructure, vegetation landscape, and availability of breeding habitats [14–18], although evidence of passive long-distance dispersal have been documented [19, 20]. A dispersal study conducted in four rural villages in Thailand demonstrated that adult *Ae. aegypti* exhibited limited dispersal, typically remaining within the same houses or houses adjacent to where they were released. Nevertheless, the same study also recorded rare long-distance movement between villages, with a maximum distance recorded at 512 m. The study further reported that the dispersal varied on the basis of the local infrastructure such as the arrangement and spacing of the houses and presence of footpaths [14]. In the temperate region of Argentina, gravid female *Ae. aegypti* were recorded laying their eggs within a range of 20–65 m, depending on the surrounding vegetation landscape [15]. A dispersal study using three model ecosystems in biosphere facility showed that *Ae. aegypti* flight distance was significantly affected by the density of breeding sites [16]. A meta-analysis of 23 mark–release–recapture (MRR) experiments conducted in tropical regions estimated that the flight distance of *Ae. aegypti* was averaged at 83.4 m [17]. A more recent meta-analysis of mean distance traveled (MDT) from 27 experiments conducted in tropical and subtropical regions reported that the weighted average MDT was 105.69 m [18].

Miami-Dade County is experiencing urban sprawl driven by an influx of residents. Rapid urban growth creates diverse aquatic habitats for mosquitoes breeding and immature development sites, which consequently affects their population dynamic and behavior [21]. Urban-driven habitat changes and population growth increase resources and vector–human contact for anthropophilic mosquitoes such as *Ae. aegypti*, potentially altering their dispersal patterns [21, 22]. Therefore, it is important to investigate the dispersal capability of *Ae. aegypti* and its associated ecological and climatic factors. The previous MRR study carried out in a residential area of Miami-Dade County estimated male *Ae. aegypti* MDTs ranging from 93 to 575 m. However, its primary aim was to characterize the dispersal ability of *Wolbachia*-infected males, and therefore, the result may not be directly relevant to other vector control programs [23].

In this study, we aimed to quantify the dispersal of both male and female *Ae. aegypti* in two different urban environments as part of a pre-intervention baseline effort. This assessment is expected to provide evidence-based guidance for local mosquito control programs in selecting and implementing larval and adult control technologies to prevent dengue outbreaks in light of ongoing urban sprawl. To achieve this, we employed mark–capture procedures involving rhodamine B self-marking units developed from rhodamine B mark–release–recapture methods [24–27]. Self-marking procedure exploits the natural behavior of mosquitoes to pick up the marker, which consequently eliminates the need for extensive handling, which could otherwise reduce mosquito fitness [28].

## Materials

### Study sites

**Fig. 1:**
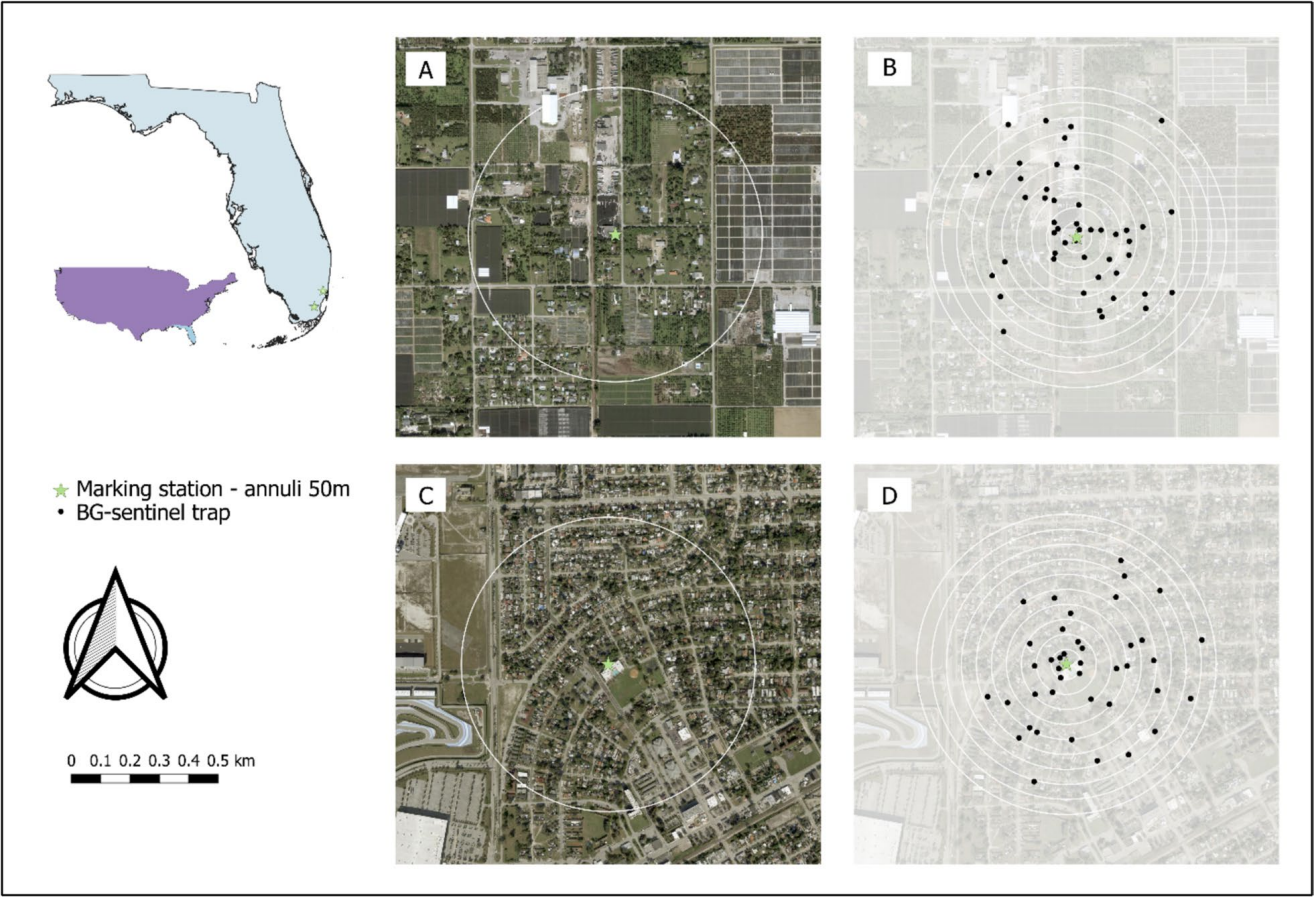
Study sites of mark–capture dispersal study in Miami-Dade County, Florida. An agribusiness district in Redlands (**A** and **B**); and a residential neighborhood in the urban area of Opa-Locka (**C** and **D**). White circles indicate concentric annuli, which were virtually drawn at 50 m intervals from the marking station

The mark–capture dispersal study was conducted in Miami-Dade County, Florida, between April and June, 2025. To account for urbanization, an agricultural area in the suburban region of Redlands and a residential area in the city of Opa-Locka were selected as study sites. The study area in Redlands is sparsely populated with high vegetation coverage [Normalized difference vegetation index (NDVI) = 0.335 ± 0.127] and is characterized by the presence of a tire yard with airplane tires [29], plant nurseries, and farms. In contrast, Opa-Locka is described as a dense urban environment with a mix of residential and commercial properties and less vegetation coverage (NDVI = 0.190 ± 0.065) compared with Redlands (Fig. 1; Additional file 1: Fig. S1).

### Rhodamine B self-marking procedure

**Fig. 2:**
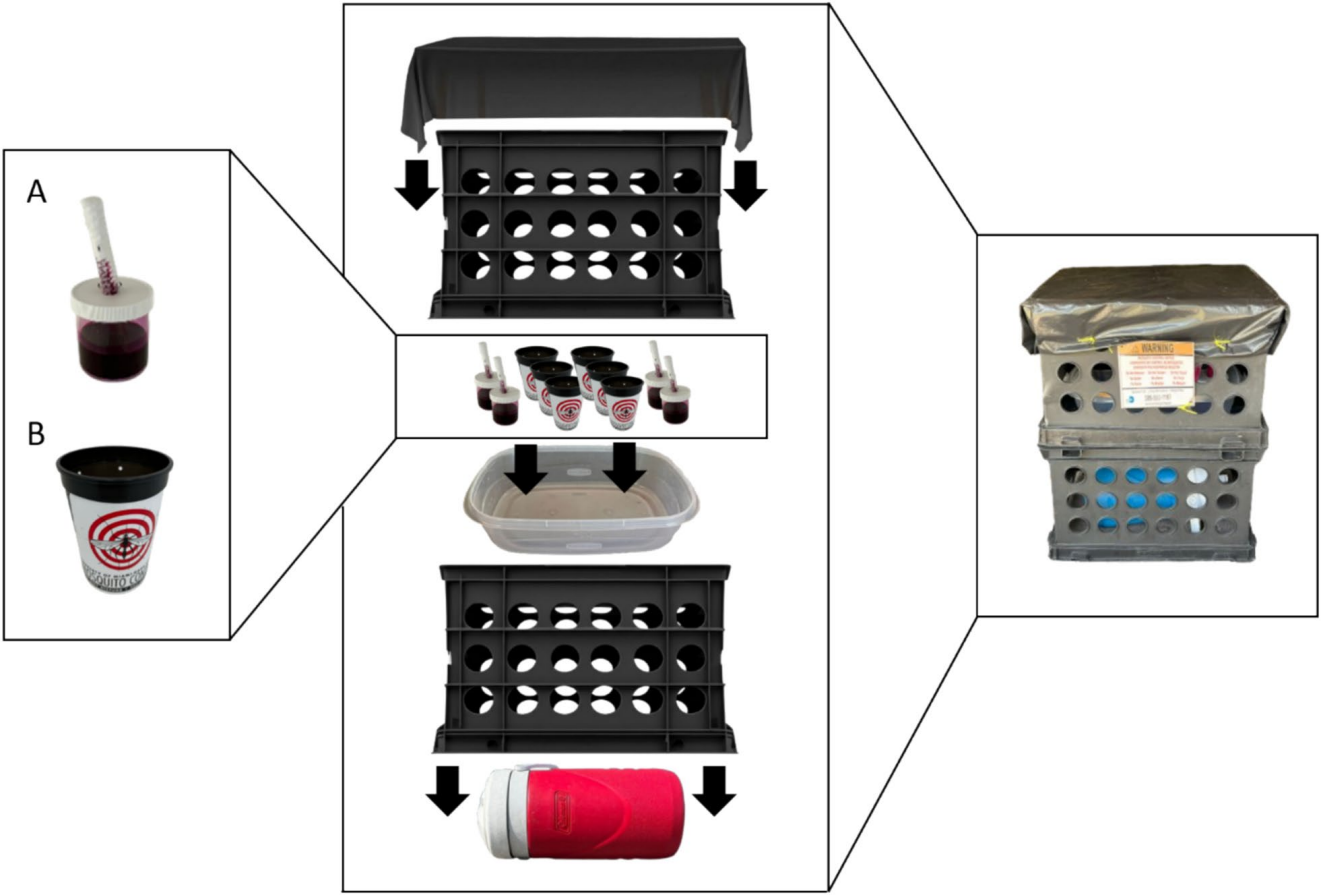
Rhodamine-B marking station. A station consists of four adult sugar feeding apparatus (**A**), six larval marking cups (**B**), a dry ice–CO_2_ lure packed in a cooler jug, and black protection structures made of crate and plastic sheeting

The marking station served as the central point for this dispersal study. In Redlands, it was situated within a 1 ha tire yard that harbored *Ae. aegypti* breeding grounds, whereas in Opa-Locka, the marking station was placed in an alley behind residential blocks. At each station, four rhodamine B marking units were installed. Each marking unit consisted of four rhodamine B sugar feeding apparatus for adult marking, six ovicups containing rhodamine B solution for larval marking, and a CO_2_ lure apparatus (a cooler jug, 1.9 L, Coleman, Wichita, KS, USA, containing 1.5 kg dry ice). All components were housed within black protective structures, built from stacked black plastic crates (39.7 × 34.9 × 26.9 cm, Sterilite, USA) and black plastic sheet (Fig. 2). Carbon dioxide and black-colored material were used to attract adult mosquitoes, thereby enhancing marking efficiency as they fed on the rhodamine B sugar solution and oviposited in the treated ovicups. To allow for adult marking, a sugar feeding apparatus with a 0.2% rhodamine B [Sigma Aldrich, 95% dye content, high-performance liquid chromatography (HPLC)] solution in 10% sugar and a braided cotton wick (Richmond Dental & Medical, Charlotte, NC, USA) were placed inside a clear plastic jar [depth (d) = 6.5 cm, height (h) = 7 cm] (Fig. 2A). The larval marking cup was a standard ovicup (d = 7.5 cm, h = 10.5 cm) lined with seed germination paper (38#, Anchor Paper Co., Saint Paul, MN, USA), filled to half of its volume with a 100 ppm rhodamine B solution (Fig. 2B). The *Ae. aegypti* larvae used for marking were collected in two ways: first, from field-collected eggs that were deposited and hatched in ovicups, and second, from natural habitats within the study site. The marking units were inspected three times a week, set on Monday, Wednesday, and Friday. The inspection included replacing or adding rhodamine B solution and CO_2_ source.

### Trapping and identification

Adult mosquitoes were collected simultaneously throughout 3-week study periods using BG-sentinel traps (Type 2, Biogents AG, Regensburg, Germany). Each BG trap was equipped with a collection net, a dry ice–CO_2_ lure apparatus and a battery (12 Volt, 12 AH, Power Sonic, Vietnam) for power supply. Net collection and trap service were scheduled for Monday, Wednesday, and Friday. In total, 48 traps in Redlands and 41 traps in Opa-Locka were deployed outdoors in circular concentric configurations at 50 m intervals, extending up to 500 m from the marking station (Fig. 1).

Collected adults were sorted by species using the mosquito identification key for North America and North Mexico [30] and sexed under a stereo microscope (SMZ745/745 T, Nikon, China). The presence of rhodamine B-marked adults was first examined using the stereo microscope and a NIGHTSEA fluorescence adapter system (NIGHTSEA SFA, Lexington, MA, USA) with cyan illumination, barrier filter (excitation 490–515 nm/emission 550 nm LP), and viewing shield. The detected rhodamine B positive adults were then confirmed using a fluorescent microscope with a DsRed filter (MZ FL III, Leica, Germany) and an X-cite 120LED illumination system (Lumen dynamic).

### Dispersal

To estimate the dispersal capacity of *Ae. aegypti*, four key metrics were calculated: minimum distance traveled (MinDT), maximum distance traveled (MaxDT), mean distance traveled (MDT), and flight range (FR). MinDT and MaxDT were determined by measuring the straight-line distance from the marking station to the nearest and farthest traps where marked mosquitoes were captured.

MDT represents the average distance from the marking point to the locations of capture, weighted by the number of mosquitoes captured at each annular distance. Both estimated capture (EC) and the correction factor (CF) are used in its calculation. Using virtual concentric annuli that were drawn within the capture area (Fig. 1), MDT was calculated using the following formula: MDT = Sum (EC × distance) for all annuli/total number of EC. The EC was defined as estimated capture: EC = (number of observed captures in annulus/number of traps in annulus) × CF. The CF accounted for differences in trap densities among annuli, hence CF = (area of annulus/total trapping area) × total number of traps. Distance is defined as inner radius plus outer radius divided by 2 [31–33].

The 50% the flight range [FR_50_] is the distance within which 50% of the marked mosquitoes were found, while the 90% flight range [FR_90_] is the distance within which 90% of the marked mosquitoes were found. Both FRs were estimated from a linear regression of cumulative ERs for each annulus plotted on the log10(distance + 1). Furthermore, FR_50_ and FR_90_ were derived from the regression equation as the distances corresponding to 50% and 90% cumulative capture, respectively.

### Weather parameters

Temperature (daily minimum and maximum) and precipitation (daily and weekly) data were obtained from the National Oceanic and Atmospheric Administration’s National Environmental Satellite, Data, and Information Service (NOAA’s NESDIS) weather stations. The stations, Homestead Gen Aviation Airport (25.5011° N, 80.5500° W) and Miami Opa-Locka Airport (25.9102° N, 80.2828° W) are approximately 6 km and 1 km away from the study site in Redlands and Opa-Locka, respectively.

### Statistical analysis

Generalized linear models (GLMs) with a negative binomial distribution [34] were used to analyze the total and marked and captured *Ae. aegypti* specimens in relation to NDVI (categorical high or low) or the presence of a tire yard (categorical yes or no), maximum (T_max_)/minimum temperature (T_min_), daily and weekly precipitation, and trap week as explanatory factors. The goodness-of-fit statistics were evaluated to ensure the model reflects the true relationship between the factors and specimen captures. The analysis was conducted using SPSS version 26.0 (SPSS Inc., Chicago, IL, USA) at α = 0.05.

A map of BG-Sentinel trap placement and virtual annuli was generated to calculate the dispersal parameters (Fig. 1). An NDVI map derived from a multispectral Landsat-8 (OLI) image (downloaded from https://earthexplorer.usgs.gov/) was generated to visualize vegetation coverage and calculate the mean of NDVI. Both maps were generated in QGIS version 3.4.15 Madeira, with the background maps obtained from Google Satellite and GADM maps and data (Creative Commons Attribution-ShareAlike 2.0 license).

## Results

### Adult trapping

**Fig. 3:**
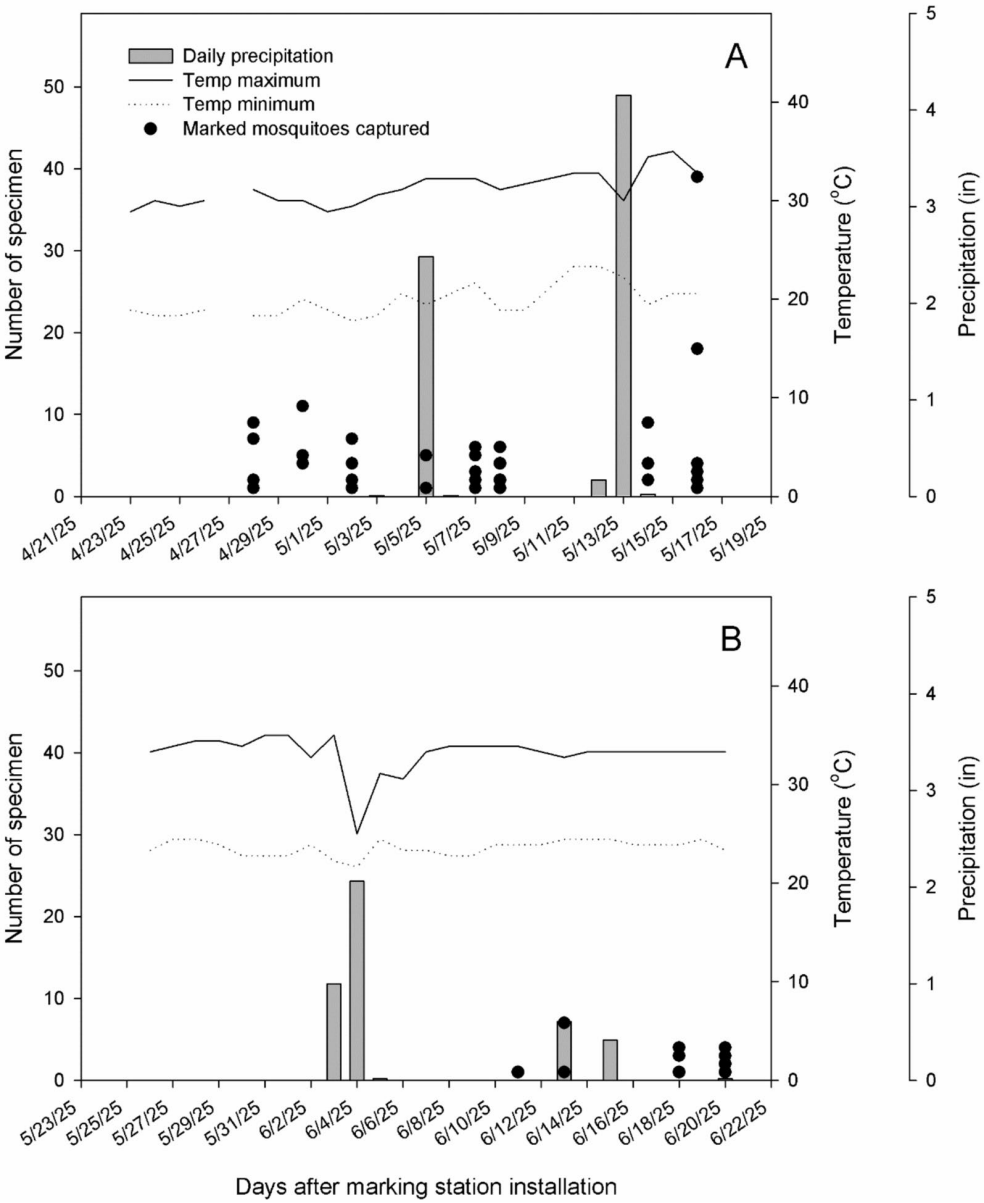
Marked *Ae. aegypti* captures, daily maximum and minimum temperatures, and daily precipitation in Redlands (**A**) and Opa-Locka (**B**) study site

During the 4-week trapping period, a total of 33,732 mosquito specimens were collected in Redlands and 17,147 specimens in Opa-Locka (Additional file 2: Dataset S1). In Redlands, *Ae. aegypti* was the predominant species, accounting for 70.1% of the total capture (23,658/33,732), followed by *Culex quinquefasciatus* (25.4%; 8580/33,732) and other culicine and anopheline species. In Opa-Locka, the species composition was largely similar, but with *Ae. aegypti* (45.4%; 7783/17,147) as the second dominant species after *Cx. quinquefasciatus* (50.9%; 8721/17,147). Among the *Ae. aegypti* collected, 216 rhodamine B–marked specimens (136 female and 80 male specimens) were captured in Redlands, while 36 rhodamine B-marked specimens (31 female and 5 male specimens) were captured in Opa-Locka. Marked *Ae. aegypti* were first detected 1 week after the installation of the marking station in Redlands and 3 weeks after the installation in Opa-Locka (Fig. 3). The proportion of marked *Ae. aegypti* relative to the total number was accounted at 3.29% ± 1.74% [(mean ± standard deviation (SD)] in Redlands and 0.66% ± 0.55% in Opa-Locka. Additionally, a few specimens from two other species, *Cx. quinquefasciatus* (nine female in Redlands; two female and three male specimens in Opa-Locka) and *Aedes albopictus* (six female and three male specimens in Redlands), were found to be marked by rhodamine B.

The total and marked *Ae. aegypti* captured were significantly impacted by the vegetation coverage or the presence of a tire yard [total: prevalence ratio (PR) = 4.93, *P* < 0.001; marked: PR = 6.17, *P* < 0.001]. Maximum temperature was not significantly associated with total specimens captured (PR = 0.95, *P* = 0.228) but was negatively associated with marked specimens captured (PR = 0.70, *P* < 0.001). In contrast, minimum temperature showed the opposite pattern, being significantly associated with the total specimens captured (PR = 1.14, *P* < 0.001) but not with marked specimens captured (PR = 1.02, *P* = 0.761). Higher daily precipitation was negatively associated with both total and marked counts (total: PR = 0.44, *P* < 0.001; marked: PR = 0.68, *P* = 0.032), whereas weekly precipitation showed no significant association with either count (total: PR = 1.48, *P* = 0.06; marked: PR = 1.46, *P* = 0.105). Moreover, the total and marked specimens collected varied by trap week, with higher counts observed during the later weeks (Table 1; Fig. 3).

**Table 1:** Results of the GLM of the total and marked *Ae. aegypti* specimens captured in relation to NDVI or presence of tire yard, maximum/minimum temperature, precipitation, and trap week as explanatory factors. Categorical variables in parentheses were used as the reference

*Ae. aegypti* captured	Statistical value	PR	95% CI	*P*-value
Total specimen count				
NDVI (low)/tire yard (no)^*^	45.84	4.93	3.11–7.83	< 0.001
T_max_	1.45	0.95	0.88–1.03	0.228
T_min_^*^	13.36	1.14	1.06–1.22	< 0.001
Daily precipitation^*^	91.96	0.44	0.37–0.52	< 0.001
Weekly precipitation	0.39	1.48	0.98–2.24	0.06
Week 1 (week 4)^*^	48.26	0.39	0.30–0.51	< 0.001
Week 2 (week 4)^*^	206.68	0.13	0.10–0.18	< 0.001
Week 3 (week 4)^*^	68.71	0.35	0.27–0.45	< 0.001
Marked specimen count				
NDVI (low)/tire yard (no)^*^	11.54	6.17	2.16–17.62	0.001
T_max_^*^	11.34	0.70	0.56–0.86	0.001
T_min_	0.09	1.02	0.87–1.21	0.761
Daily precipitation^*^	4.60	0.68	0.47–0.97	0.032
Weekly precipitation	0.38	1.46	0.92–2.32	0.105
Week 1 (week 4)	n.a.	n.a.	n.a.	n.a.
Week 2 (week 4)^*^	21.66	0.17	0.08–0.36	< 0.001
Week 3 (week 4)^*^	21.62	0.32	0.19–0.51	< 0.001

*PR* prevalence ratio, *CI* confidence interval, *n.a.* not available, *GLM* generalized linear model

^*^Results are statistically significant at α = 0.05

### Dispersal

**Fig. 4:**
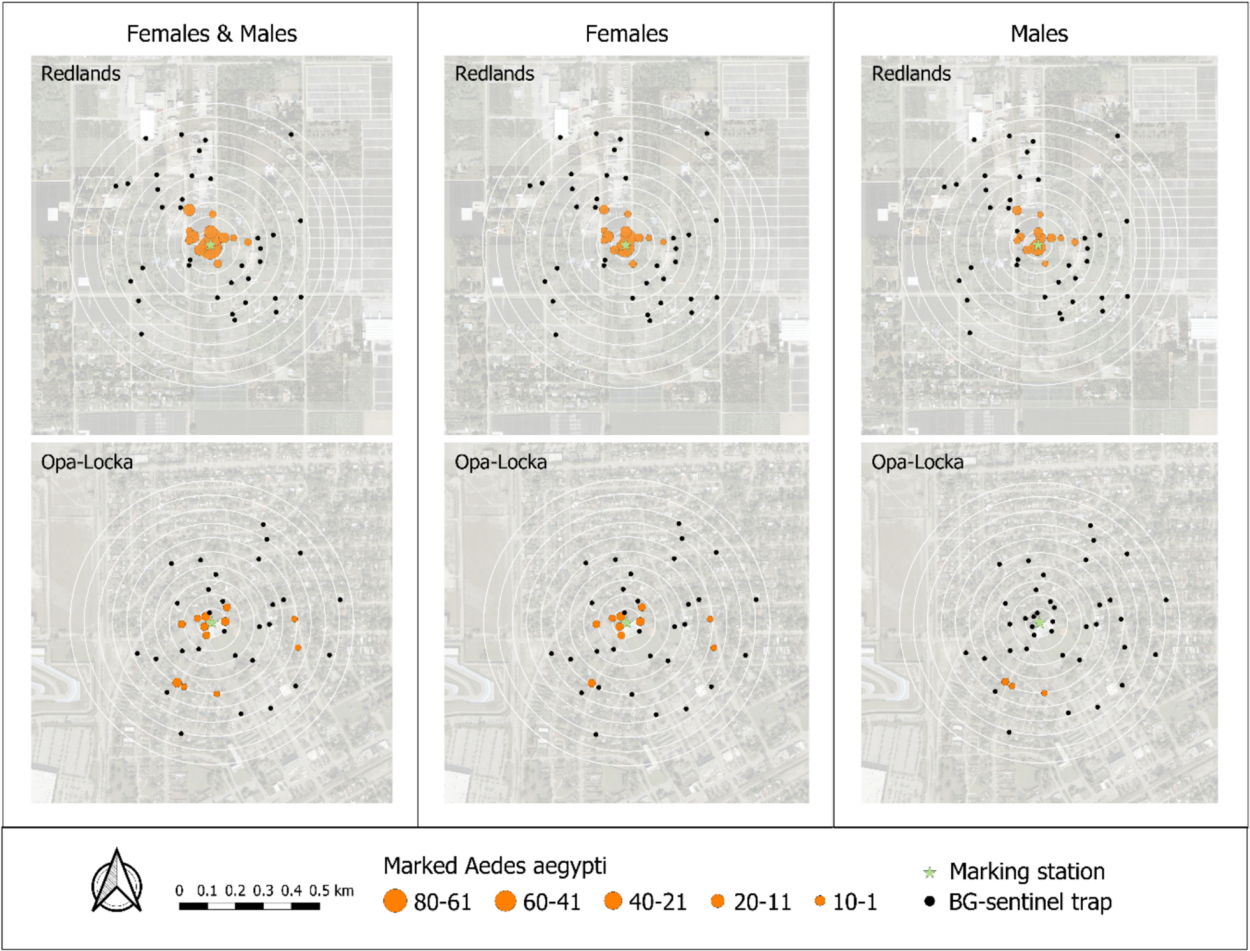
The density of rhodamine B positive *Ae. aegypti* in the Redlands and Opa-Locka study site

In Redlands, 71.3% (154/216) of marked *Ae. aegypti* were found primarily within a radius of 50 m, followed by 18.0% (41/216) within a radius of 100 m, and 9.7% (21/216) within a radius of 150 m. No marked specimens were collected beyond a radius of 150 m (Fig. 4). The marked mosquitoes had an MDT of 63.65 m, with a MinDT of 10.07 m and a MaxDT of 144.28 m. The overall FR_50_ and FR_90_ in Redlands were estimated to be 24.99 m and 171.21 m, respectively (Table 2).

**Table 2:** Dispersal parameters of rhodamine B marked *Ae. aegypti* in each study site, Redlands and Opa-Locka, Miami-Dade County, Florida, USA

Study site	Sex	Min/MaxDT (m)	MDT (m)	FR_50_ (m)	FR_90_ (m)
Redlands	Female	10.07/144.28	60.66	21.25	165.45
	Male	10.07/144.28	68.33	31.57	180.19
	Both	10.07/144.28	63.65	24.99	171.21
Opa-Locka	Female	28.60/316.29	179.51	114.52	334
	Male	245.12/251.04	235.38	169.52	309.21
	Both	28.60/316.29	194.03	131.83	329.58

In comparison to Redlands, Opa-Locka marked specimens were detected at greater distances, with collections extending up to 350 m from the marking station. The overall MDT was estimated to be 194.03 m with a MinDT of 28.60 m and a MaxDT of 316.29 m. Furthermore, 50% of marked specimens were dispersed within 131.83 m (FR_50_), while 90% dispersed within 329.58 m (FR_90_).

Dispersal parameters calculated by sex for both study sites are presented in Table 2, and the density of marked mosquitoes per trap is depicted in Fig. 4. In Redlands, the MinDT and MaxDT were 10.07 m and 144.28 m for both females and males. In Opa-Locka, females had a MinDT of 28.60 m and a MaxDT of 316.29 m, whereas males showed a MinDT of 245.12 m and a MaxDT of 251.04 m. Mean distances traveled (MDT) were consistently lower for females than for males at both sites, measuring 60.66 m and 68.33 m in Redlands and 179.51 m and 235.38 m in Opa-Locka for females and males, respectively. In Redlands, 50% of marked females and males were recovered within 21.25 m and 31.57 m, whereas in Opa-Locka they were recovered within 114.52 m and 169.52 m. Likewise, 90% of marked females and males were found within 165.45 m and 180.19 m in Redlands and within 334 m and 309.21 m in Opa-Locka.

## Discussion

We quantified the dispersal of both sexes of *Ae. aegypti* in Redlands and Opa-Locka, representing two different urban landscapes in Miami-Dade County. Measuring mosquito vector dispersal capability and its associated factors is crucial prior to designing vector control interventions aimed at achieving a more accurate delineation of spatial coverage. Our data showed that the trapping yield of marked specimens was influenced by the urban ecological and climatic conditions, including the presence of high/low vegetation coverage or a tire yard as prolific breeding habitats, temperature, and precipitation. These factors may have contributed to the differences in dispersal distance and flight range of *Ae. aegypti* between the two study sites.

### Evaluation of self-marking procedure

Unlike conventional MRR, the self-marking procedure allows the inclusion of female mosquitoes in dispersal estimates without increasing vectorial capacity or nuisance biting compared with the undisturbed natural baseline [35, 36]. Self-marking can also reduce potential bias in the results caused by direct handling of mosquitoes [12, 28]. However, these procedures have limitations, as they cannot be used to estimate wild mosquito population size, as all of the formulae require the total number of marked mosquitoes released [37]. Estimating vector population size is part of the pre-intervention baseline data, which is particularly essential to measure the effectiveness of intervention methods. Consequently, the self-marking procedure using naturally occurring mosquitoes has generally been limited to investigating dispersal abilities and movement patterns [11, 13, 38].

Our marking station was designed to optimize mosquito exposure to rhodamine B via a sugar feeding apparatus and rhodamine B solution in ovicups (Fig. 2). We provided a source of sugar and equipped the marking station with host-seeking and oviposition cues, including a black-shady structure, CO_2_, and an oviposition site [39]. These elements were intended to simulate natural feeding, resting, and breeding conditions, thereby attracting female and male mosquitoes to visit the station and increase the possibility of successful adult marking. Adult marking may have also occurred through newly eclosed adults emerging from the ovicups since the adult typically seeks sugar for their first meal [40], especially when the larvae are undernourished [41]. This behavior facilitates early ingestion of the dye. Regarding larval marking, we did not observe any *Aedes* eggs on the ovicup substrate; instead, several *Culex* egg rafts were found floating on the water surface inside the ovicup, which were removed immediately. Using ovicups filled with 100 ppm rhodamine B solution to collect *Aedes* eggs as a source for larval marking proved to be challenging in the selected sites. To overcome this limitation, *Aedes* larvae found within the study sites were collected and transferred to the marking station. However, the collected larvae varied in their developmental instar, which may affect the detectability of rhodamine B in adults under fluorescence microscopy. Although the successful marking rate could not be quantified, the current rhodamine B self-marking technique resulted in marked mosquito capture rates ranging from 0.66% to 3.29%. In comparison, the enrichment rate of a self-marking technique using stable isotopes was estimated to be 2.12–11% [13], whereas another study reported recapture rates of enriched mosquitoes ranging from 0.23% to 0.52% [11]. The effectiveness of the marking technique was further supported by the capture of marked nontarget species (*Cx. quinquefasciatus* and *Ae. albopictus*), which are also container-breeding mosquitoes.

Vegetation coverage, the presence of a tire yard as prolific breeding habitats, and daily precipitation emerged as the major drivers influencing the total and marked *Ae. aegypti* captured in this mark–capture study (Table 1). This finding is consistent with previous studies in Miami-Dade County, which reported that vegetation coverage was positively correlated with mosquito abundance [4] and that tire shops served as an important source of vector mosquitoes [42]. In this study, lower mosquito abundance means lower marked mosquito catchment and a longer lag time before marked mosquitoes were detected, as observed in Redlands and Opa-Locka (Table 1; Fig. 3). This result suggests that the rhodamine B self-marking procedure may have limited effectiveness in setting where the target mosquito population is low. Furthermore, this study found that the daily rainfall was negatively associated with mosquito captures. Lower captures during rainy days were likely due to a combination of factors, such as changes in mosquito behavior [43], increased mortality caused by rainfall events [44], and decreased trap efficacy under wet conditions [45]. This finding suggests that precipitation should be considered an important covariate in mark–capture dispersal studies, including weekly cumulative precipitation, which may favor mosquito captures, although this relationship was not statistically significant in the present study.

Although wind speed, wind direction, and humidity may influence the short-range dispersal of *Ae. aegypti* [26, 46, 47] and adult trapping efficiency [48, 49], wind and humidity variables were not included in this present analysis. The exclusion was due to the lack of reliable high-resolution wind data synchronized spatially and temporally with marking stations and trap locations for both study sites. Future studies incorporating microclimate sensors could further elucidate the role of wind and humidity on *Ae. aegypti* dispersal in urban environments.

### Dispersal

The MDT values calculated in both study sites (Redlands, 63.65 m; Opa-Locka, 194.03 m) fell within the range of those reported in a meta-data analysis of *Ae. aegypti* flight distances from various regions (30.50–199.00 m, median = 105.69 m) [15]. Aside from the dispersal study conducted in Miami-Dade County [23], only a few other *Ae. aegypti* dispersal studies have been reported in the USA. In residential areas surrounded by agricultural fields in South Texas, the MDT of a field population marked with stable isotopes ranged between 220 and 255 m for males, 135–189 m for gravid females, and 192–213 m for unfed females [13]. In an urbanized area within a semi-arid climate in Central California, the MDT of a stable isotope-labeled colony was estimated between 224 and 240 m [49]. In the project of sterile insect technique (SIT) on Captiva Island, a barrier island off Florida’s southwest coast, the MDT of sterile males was estimated around 201.7 m [50]. Our results show that the dispersal of *Ae. aegypti* varies depending on the urban landscape, providing more information for designing more effective intervention strategies in the USA.

Comparatively, the dispersal capacity of *Ae. aegypti* was greater in the residential area of Opa-Locka than in Redlands (Table 2). In the Redlands study site, the marking station was placed within a tire yard surrounded by plant nurseries, farms, and shrubs. In contrast, the Opa-Locka marking station was situated within a residential area characterized by typical housing blocks, where breeding sources were more widely scattered. Our findings indicate that, *Ae. aegypti* dispersal is highly localized when mates, blood sources, sugar sources, and breeding sites are all readily available nearby, similar to a study reported by Harrington et al. [14]. Brown et al. reported that, *Ae. aegypti* dispersal was influenced by the interaction between oviposition site density and ecosystem type [16]. When oviposition sites were sparse, the dispersal was found to be greater in the vegetation-rich humid rainforest than in the vegetation-poor, arid desert-type ecosystem. In our highly vegetated study area (Redlands), oviposition sites were not sparse but rather were concentrated within the tire yard. In contrast, the Opa-Locka study site featured typical man-made oviposition sites characteristic of an urban environment. The density and spatial distribution of oviposition sites may partially explain the observed differences in dispersal capability between Redlands and Opa-Locka.

The estimated dispersal of females was fairly comparable to that of males in Redlands, but considerably lower in Opa-Locka (except for FR_90_) (Table 2; Fig. 4). According to Moore and Brown, sex was not associated with MDT [18]. However, lower female MDT compared with male MDT was observed in an MRR study in residential areas of South Texas [13]. Our finding highlights the importance of future studies aimed at identifying the major factors impacting movement in female and male mosquitoes. Active mosquito movement is known to be driven by the search for blood sources, sugar sources, mates, oviposition sites, and resting sites or shelters [17, 46]. Nevertheless, specific factors that differentially determine movement in females versus males remain insufficiently explored. Understanding male dispersal is now considered to be as important as that of females since many innovative control strategies rely on males to suppress vector populations [51–53].

### Implications for the vector control strategy

For decades, the Miami-Dade Mosquito Control Division has been actively conducting county-wide public outreach activities, mosquito population surveillance, and control activities in response to citizen reports and referrals from the Department of Health of suspected cases of mosquito-borne diseases. The control measures currently implemented by the division include breeding site removal (i.e., drain and cover campaigns, storm drain flushing), larvicide applications [i.e., *Bti*, insect growth regulator (IGR), and larvivourus fish], and adulticide applications (i.e., handheld, truck, and aerial space spraying). Information on dispersal obtained from our study may provide valuable technical guidance to mosquito control programs in planning insecticide-based interventions. Because *Ae. aegypti* dispersal is limited, control operations can be more localized and precisely targeted through combined focal space spraying and larviciding [12, 13]. In more detail, our findings in a semiurban agriculture environment such as Redlands suggest that insecticide space spraying could be effectively concentrated within a 100 m radius around premises harboring *Ae. aegypti* larval breeding sites.

The findings from this study are particularly valuable should mosquito control decide to implement additional intervention techniques. In density-dependent adult control measures such as attractive toxic sugar baits (ATSB) [54] and mass trapping [55], dispersal data can inform the optimal density and spatial arrangement of baiting or trapping units to maximize effectiveness. Moreover, the vector dispersal dynamics represent a critical factor in establishing release strategies for rear-and-release intervention technologies, such as the deployment of *Wolbachia*-infected mosquito and sterile insect technique. Understanding how far and in what patterns *Ae. aegypti* (mainly male) disperse informs decisions on release density, release frequency, and spatial distribution, ensuring that released mosquitoes effectively mix with wild populations. Inadequate consideration of dispersal behavior could lead to suboptimal coverage, reinfestation from surrounding untreated areas, or inefficient use of resources, ultimately reducing the success of such interventions [18, 46].

## Conclusions

This study evaluated a rhodamine B self-marking method for application in a mark–capture procedure to assess dispersal of the local population of *Ae. aegypti* in two diverse urban environments in Miami-Dade County, Florida. Marked mosquitoes were successfully detected in both study sites, although the timing and proportion of captures varied depending on local environmental conditions, such as vegetation cover, the presence of a tire yard as major breeding habitats, and precipitation. Dispersal distances were greater in the residential setting of Opa-Locka than in the semiurban agricultural environment of Redlands. This finding suggests that anthropogenic features in highly urbanized landscapes play an important role in mosquito dispersal and may differentially influence the dispersal of females and males. The dispersal distances and flight ranges estimated in this study were broadly comparable to those reported in previous *Ae. aegypti* dispersal studies conducted in the USA. The study outcomes provide valuable evidence for guiding spatially targeted mosquito control strategies in urban environments.

## Supplementary Information

Supplementary Material 1. Fig. S1. Normalized Difference Vegetation Index (NDVI) classification in Redlands (**A**) and Opa-Locka (**B**) study sites.

Supplementary Material 2. Dataset S1. Adult trapping and capture

## Data Availability

The data sets supporting the conclusions are available in this article and supplementary data.

## Abbreviations

MinDT: Minimum distance traveled
MaxDT: Maximum distance traveled
MDT: Mean distance traveled
FR: Flight range
MRR: Mark–release–recapture
NDVI: Normalized difference vegetation index
EC: Estimated capture
CF: Correction factor
GLMs: Generalized linear models
NOAA: The National Oceanic and Atmospheric Administration
OLI: Operational land imager
DT: Distance traveled
*Bti*: *Bacillus thuringiensis israelensis*
IGR: Insect growth regulator
ATSB: Attractive toxic sugar bait
