# Supplementary material for: Dispersal of Aedes aegypti in urban environments of Miami-Dade County, Florida
Source: Parasit Vectors. 2026 May 18;19:285. doi: 10.1186/s13071-026-07445-7 (PMC13349064; doi:10.1186/s13071-026-07445-7)
Supplement: Supplementary file 1 — Supplementary Material 1. Fig. S1. Normalized Difference Vegetation Index (NDVI) classification in Redlands (A) and Opa-Locka (B) study sites. [file 13071_2026_7445_MOESM1_ESM.docx]

**Additional File 1**


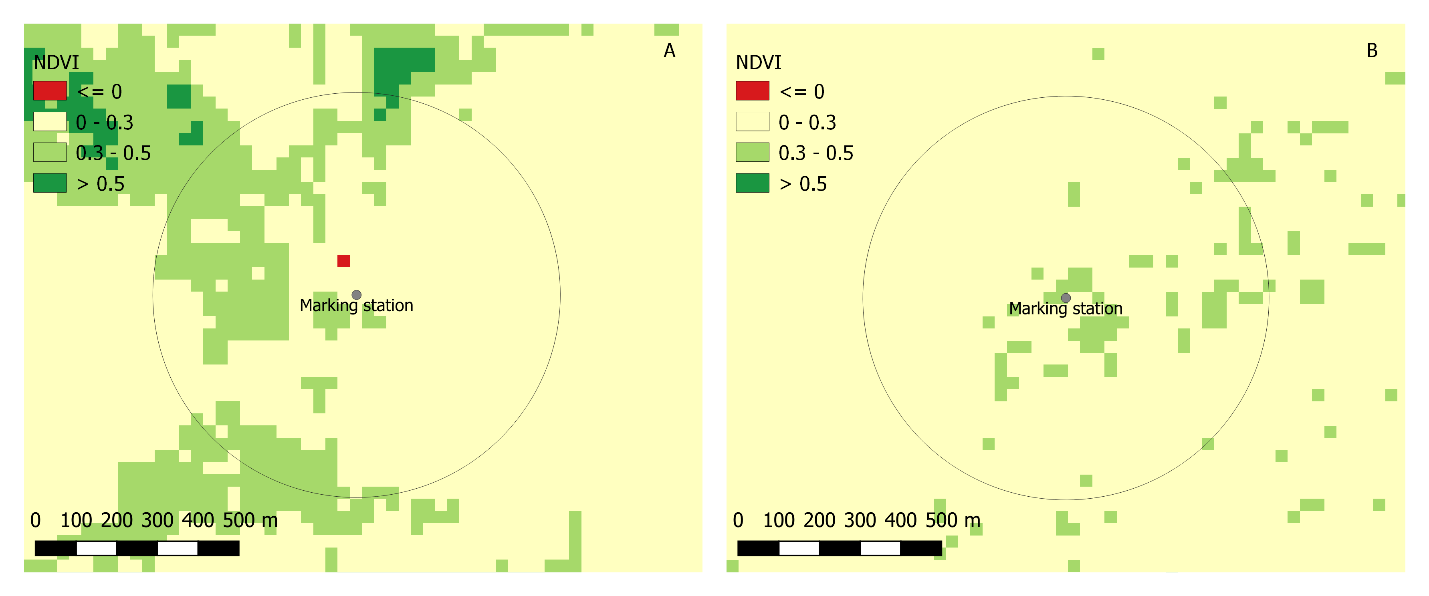


Fig. S1. Normalized Difference Vegetation Index (NDVI) classification in Redlands (A) and Opa-Locka (B) study sites.
